# Polyherbal dietary supplementation for prediabetic adults: study protocol for a randomized controlled trial

**DOI:** 10.1186/s13063-018-3032-6

**Published:** 2019-01-07

**Authors:** Termeh Feinberg, L. Susan Wieland, Larry E. Miller, Kashif Munir, Toni I. Pollin, Alan R. Shuldiner, Steve Amoils, Lisa Gallagher, Mary Bahr-Robertson, Christopher R. D’Adamo

**Affiliations:** 1 University of Maryland School of Medicine Center for Integrative Medicine, Baltimore, MD USA; 2 Yale University School of Medicine Center for Medical Informatics, New Haven, CT USA; 3 Miller Scientific Consulting, Asheville, NC USA; 4 University of Maryland School of Medicine Center for Diabetes and Endocrinology, Baltimore, MD USA; 5 University of Maryland School of Medicine Department of Medicine, Baltimore, MD USA; 6 Alliance Integrative Medicine, Cincinatti, OH USA

**Keywords:** Prediabetes, Botanical, Berberine, Fenugreek, American ginseng, Gymnema, Banaba, Kudzu, Cinnamon, Glucose metabolism disorders

## Abstract

**Background:**

Prediabetes describes a state of hyperglycemia outside of normal limits that does not meet the criteria for diabetes diagnosis, is generally symptomless, and affects an estimated 38% of adults in the United States. Prediabetes typically precedes the diagnosis of type 2 diabetes, which accounts for increased morbidity and mortality. Although the use of dietary and herbal supplements is popular worldwide, and a variety of single herbal medicines have been examined for glycemic management, the potential of increasingly common polyherbal formulations to return glycemic parameters to normal ranges among adults with prediabetes remains largely unexplored. The purpose of this study is to evaluate the efficacy of a commercially available, polyherbal dietary supplement on glycemic and lipid parameters in prediabetic individuals.

**Methods:**

In this multi-site, double-blinded, randomized controlled clinical trial, 40 participants with prediabetes will be randomized to either a daily oral polyherbal dietary supplement (GlucoSupreme™ Herbal; Designs for Health®, Suffield, CT, USA; containing cinnamon bark (*Cinnamomum cassia*), banaba leaf (*Lagerstroemia speciosa* standardized to 1% corosolic acid), kudzu root (*Pueraria lobata* standardized to 40% isoflavones), fenugreek seed (*Trigonella foenum-graceum* standardized to 60% saponins), gymnema leaf (*Gymnema sylvestre* standardized to 25% gymnemic acid), American ginseng root (*Panax quinquefolius* standardized to 5% ginsenosides), and berberine HCl derived from bark (*Berberis aristata*)) or placebo for 12 weeks. Short-, medium-, and comparatively long-term markers of glycemic control (blood glucose and fasting insulin, fructosamine, and glycated hemoglobin/A1c, respectively), and other glycemic parameters (GlycoMark, β-cell function, and insulin sensitivity/resistance) will be obtained. Lipid profile (total cholesterol, LDL, HDL, and triglycerides), inflammation (hs-CRP), progression to type 2 diabetes mellitus, as well as safety indices (ALT, AST) will be obtained. An intention-to-treat analysis will be used to assess changes in study outcomes.

**Discussion:**

Treatment options for adults with prediabetes are currently limited. This study aims to evaluate the safety and efficacy of a commercially available dietary supplement in the popular, but as yet insufficiently studied, category of polyherbal formulas for the management of glycemic parameters and other biomarkers associated with prediabetes.

**Trial registration:**

ClinicalTrials.gov, ID: NCT03388762. Retrospectively registered on 4 January 2018.

**Electronic supplementary material:**

The online version of this article (10.1186/s13063-018-3032-6) contains supplementary material, which is available to authorized users.

## Background

Prediabetes, a disorder for which global prevalence is rapidly increasing [1], affects 38% of people in the United States [2]. People with prediabetes present with slightly elevated glucose levels (i.e., fasting plasma glucose between 100 and 125 mg/dL, glycated hemoglobin/A1c (HbA_1c_) between 5.7 and 6.4%, or postprandial glucose between 140 and 199 mg/dL), but not high enough to be considered type 2 diabetes (T2D) [3]. People with prediabetes often do not display physical symptoms; however, prediabetes perpetuates pancreatic β-cell dysfunction [4] and nearly always precedes diagnosis of T2D [3], a disease affecting approximately 9% of adults in the United States [5]. Prediabetes and T2D is often accompanied by comorbid low HDL (high-density lipoprotein) cholesterol and high triglycerides, and is responsible for increased risk of macrovascular disease such as heart disease and stroke. T2D further increases risk for microvascular disease such as retinopathy (which may lead to blindness and other visual problems), nephropathy (which may lead to kidney failure), neuropathy (a leading cause of amputation), and death [2, 6, 5].

Current recommendations for managing prediabetes and preventing progression to T2D remain limited due to very few pharmacological options with favorable safety profiles [7, 8] and the challenges posed in successful implementation and maintenance of certain lifestyle modifications (i.e., increased physical activity, diet, and weight loss) [3, 9].

A number of herbs have been used to manage symptoms related to deleterious changes in glucose metabolism [10–17]. A number of clinical studies of herbal interventions (i.e., cinnamon, fenugreek, banaba, curcumin, and others) conducted among adult populations with prediabetes have demonstrated improved glycemic control, particularly with regard to reducing fasting blood glucose [18–25] and postprandial glucose [18, 22, 26–29], HbA_1c_ [25, 30, 31], fasting insulin levels [9, 26, 28–31], homeostatic model of Insulin Resistance (HOMA-IR) [20, 28, 30, 32, 33], and increasing homeostatic model of β-cell function (HOMA-β) [27, 32]. Substantial changes in glycemic parameters typically occur within 12 weeks [34, 35]. Likewise, each of these outcomes have demonstrated significant changes over a 12-week duration in previous trials [19, 20, 22, 28, 29, 33, 36].

Several herbs have also demonstrated benefit in glycemic control in clinical trials among T2D populations. These herbs and herbal extracts include berberine [37–39], ginseng [40, 41], gymnema [42, 43], banaba [44], cinnamon [24, 45], fenugreek [46], and kudzu [47]. Of these, some have also demonstrated potential benefit for promoting positive changes in lipid markers [37, 48–60].

Each of these seven plant ingredients may improve glycemic control, and all have a wide history of ethnobotanical use in raw unprocessed, crudely processed, or other preparations [15, 17, 61]. Approximately 80% of the total content of free amino acids in fenugreek seeds is represented by 4-hydroxyisoleucine, and appears to directly stimulate insulin in a glucose-dependent manner [62–65]. Likewise, in vitro research suggests that some active constituents of the banaba leaf have an insulin-like effect [66, 67]. Lagerstroemin, an ellagitannin present in banaba, may activate insulin receptors [44, 65, 66, 68]. In addition, kudzu likely increases glucose utilization [69] and activates alpha-1 adrenoceptors in the adrenal gland to enhance the secretion of beta-endorphin, resulting in decreased plasma glucose [70]. Gymnema leaves contain oleanane triterpene saponins, which include gymnemic acids [71]. Gymnemic acids appear to reduce intestinal absorption of glucose and may stimulate pancreatic β-cell growth [72, 73]. Gymnema may also have a direct effect on β-cell function, thus resulting in the release of insulin [74]. Further, gymnema may also increase serum C-peptide levels, indicative of a potential increase in endogenous insulin secretion [43].

Other plant ingredients in GlucoSupreme™ Herbal, a commercially available polyherbal dietary supplement, may be responsible for similar mechanisms of action. Polyphenolic polymers, found in cinnamon, appear to potentiate insulin action by increasing phosphorylation of the insulin receptor, thereby increasing insulin sensitivity, which may lead to improvements in blood glucose control and lipid levels. Cinnamon extracts also appear to activate glycogen synthetase and increase glucose uptake [75–77]. Berberine, another ingredient present in the study treatment, stimulates glucose transport through a mechanism distinct from insulin via increasing GLUT1 activity [78], and improves glucose metabolism through induction of glycolysis related to the inhibition of glucose oxidation in mitochondria. Berberine-induced AMPK activation is likely a consequence of mitochondrial inhibition that increases the AMP/ATP ratio [38]. Thus, at least some of berberine’s potential effects in the treatment of diabetes derive from stimulation of AMPK activity [79]. Berberine also enhances signaling, notably through increased glucose transporter-4 translocation into the plasma membrane via the enhancement of insulin signaling pathways and insulin receptor substrate-1-phosphoinositide 3-kinase-Akt. In addition, berberine appears to increase glucose-stimulated insulin secretion and proliferation in Min6 cells via an enhanced insulin/insulin-like growth factor-1 signaling cascade. Berberine likely decreases glucose absorption through the intestinal epithelium in part due to its ability to inhibit alpha-glucosidase [80], and may act as an effective insulin-sensitizing and insulinotropic treatment [81]. The chemical structure of berberine and related isoquinoline alkoloids differs from other commonly used hypoglycemic agents, such as sulfonylureas, biguanides, thiazolidinediones (TZDs), and acarbose [38].

Ginsenosides are triterpenoid saponins and the major active constituents of ginseng [82], and target different types of tissues, producing metabolic and other pharmacological responses [40, 83]. Antidiabetic effects demonstrated by ginsenosides present in American ginseng root, including Rb_1_ [40, 83, 84], are likely related to their ability to activate peroxisome PPAR-γ, which is responsible for regulation of expression of key genes involved in lipid and glucose metabolism and adipocyte differentiation [83, 85, 86]. The activation of PPAR-γ causes body-wide lipid repartitioning by increasing the triglyceride content in adipose tissue and lowering free fatty acids in circulation, liver, and muscle, thus leading to improvements in insulin sensitivity [40, 83, 87]. Likewise, the transcriptional response of PPAR-γ may result in the recruitment of cofactors which increase insulin-stimulated glucose uptake, and positively regulate glucose metabolism and energy expenditure [83, 88, 89].

TZDs are full PPAR-γ agonists [90] often prescribed in the clinical treatment of T2D, but often result in undesirable side effects [83, 91, 92]. Alternately, the potential antidiabetic effects of the partial PPAR-γ agonist ginsenoside Rb_1_ is exemplified through the promotion of adipocyte differentiation via PPAR-γ activation and increased activation of glucose transporter 4 (GLUT4) associated with insulin sensitivity in adipocyte tissue [83, 84, 87]. Therefore, American ginseng may improve insulin resistance by reducing lipotoxicity in muscle and liver through increasing the ability to store lipids in adipocytes, and enhance insulin sensitivity through increase of adipocyte GLUT4 expression [83].

While many of these herbs appear to be promising when used in isolation, the practice of herbal medicine most often utilizes polyherbal combinations for purported synergistic effects [82]. Although a handful of polyherbal dietary supplement formulations [9, 20, 21, 33, 93, 94] have been examined for the improvement of glycemic control among prediabetic populations, no study has yet examined the potential impacts of berberine, ginseng, gymnema, banaba, cinnamon, fenugreek, and kudzu as a polyherbal formulation using a comprehensive panel of validated markers of lipid status and glycemic control (i.e., short-term, medium-term, and longer-term) utilized in both research and clinical practice.

In light of the vast number of commercially available, polyherbal formulas that have not yet been studied, rigorous clinical trials are needed to evaluate the efficacy of commercially available, polyherbal dietary supplements. This remains an especially important research gap since the dosages and ingredients in these formulas are highly heterogeneous and have not been well-examined.

## Methods

### Objectives

The primary objective of this study is to evaluate the impact of a commercially available, polyherbal dietary supplement on markers of glycemic control among a sample of adults with prediabetes. The secondary objectives are to investigate the effects of this supplement on lipid and inflammatory markers.

### Research type

A 12-week, randomized, double-blinded, placebo-controlled clinical trial is being conducted to achieve the objectives of this study. The authors hypothesize that GlucoSupreme™ Herbal will improve a variety of validated markers of glycemic control more effectively than placebo. In accordance with previous studies of dietary supplements among prediabetic adults and the interval between measurements of glycemic control in clinical practice, this proof-of-concept and exploratory study is 12 weeks in duration.

### Participants

#### Screening of participants

Key entry criteria are adults who satisfy one or more American Diabetes Association (ADA) criteria for prediabetes, and do not include those diagnosed with diabetes. Among other criteria provided in Table 1, daily tobacco smokers were excluded due to unknown safety interactions with nutritional components in the study treatment. Previous research demonstrated an increased risk of lung cancer in smokers consuming β-carotene dietary supplements [95], and β-carotene is naturally present and sometimes highly abundant in many plants, including kudzu [96].

**Table 1:** Inclusion and exclusion criteria

Critera	
Inclusion	Exclusion
Aged ≥18 years Fulfill prediabetes’ diagnostic criteria, determined by blood measurement values obtained within past 12 weeks Agree to continue with current diet and refrain from taking any new nutritional or herbal supplements during the study Agree to continue with current physical activity level throughout study period Able to understand and write English Voluntarily consent to the study and understand its nature and purpose including potential risks and side effects	Current daily use of any oral hypoglycemic medication or insulin injection Any current or previous diagnosis of diabetes (type 1 or type 2) Current daily use of any supplement containing the herbs in the study supplement Known allergies to any substance in the study supplement Current daily tobacco smoker Currently pregnant or breastfeeding, or planning to become pregnant in the next 12 weeks Myocardial infarction, vascular surgery, or stroke in the past year Refusal to provide consent for the study

##### Diagnostic criteria

Prediabetes is classified as demonstrating any of the following, according to ADA guidelines [3, 97]:

1. Hemoglobin A1c (HbA_1c_) of 5.7–6.4%
2. Fasting blood glucose of 100–125 mg/dL
3. 2-h 75-g Oral Glucose Tolerance Test blood glucose value of 140–199 mg/dL

##### Source of participants

Study participants will be identified and recruited from community health fairs, diabetes support groups, and directly from clinicians in University of Maryland Medical System outpatient facilities (Baltimore, MD, USA), including the Center for Diabetes and Endocrinology, and Family Medicine Associates. Alliance Integrative Medicine (Cincinnati, OH, USA), and a population of Amish research participants (Lancaster, PA, USA) will serve as additional sources. A member of the research team will screen and include participants via physician referral and/or by verification of laboratory parameters (fasting plasma glucose: 100–125 mg/dL and/or HbA_1c_: 5.7–6.4% and/or postprandial glucose: 140–199 mg/dL), indicating prediabetes status within past 12 weeks, according to ADA guidelines [3]. Male and female participants will be eligible to participate. Based on the racial and ethnic diversity across all sites, a racially and ethnically diverse study population is anticipated.

### Intervention

#### Arrangement for intervention

This study is a multi-site clinical trial, with treatment allocation randomized a priori within each site. This study has received Institutional Review Board (IRB) approval. All research staff have experience with clinical trial recruitment, consenting procedures, and management of clinical and community interventions, and have been trained to uniformly implement all procedures relevant to this study prior to participation. After providing informed consent, participants are instructed on the details of their involvement in the study, including how to use their daily dietary supplement (or placebo) and study diary. Each participant has an in-person baseline assessment, a telephone assessment at 6 weeks, and an in-person assessment at 12 weeks.

#### Polyherbal dietary supplement intervention

GlucoSupreme™ Herbal (Designs for Health®, Suffield, CT, USA) is a polyherbal formula for glycemic control which contains many of the herbs and their standardized extracts that have been previously studied and shown to be potentially efficacious for glycemic control [24, 37–47]. This supplement has been commercially available in the United States since 2009. Each daily serving of four GlucoSupreme™ Herbal capsules includes extracts from: contains cinnamon bark (*Cinnamomum cassia*) 500 mg, banaba leaf (*Lagerstroemia speciosa* standardized to 1% corosolic acid) 200 mg, kudzu root (*Pueraria lobata* standardized to 40% isoflavones) 200 mg, fenugreek seed (*Trigonella foenum-graceum* standardized to contain 60% saponins) 200 mg, and gymnema leaf (*Gymnema sylvestre* standardized to contain 25% gymnemic acid) 200 mg. Additionally, the supplement contains American ginseng root (*Panax quinquefolius* standardized to contain 5% ginsenosides) 200 mg, and berberine HCl derived from bark (*Berberis aristata*) 500 mg. The study treatment is the suggested dosage of this product, and the dosages of active ingredients are comparable or less than those administered in previously conducted clinical studies [20, 22, 37–40, 42, 43, 98–105].

The study sponsor will provide verification data that active study supplement batches contain ingredients as indicated. Other ingredients include cellulose (capsule), microcrystalline cellulose, silicon dioxide, and vegetable stearate. Participants are advised to take each two-capsule serving of GlucoSupreme™ Herbal with food, twice per day (the suggested amount advertised on commercially available bottles), for 12 weeks.

All participants visit their clinic site for two total visits over the 12-week study (i.e., baseline and at 12 weeks). Each visit includes blood sampling. Participants receive their study supplement from the same outpatient physician office, health clinic, or at a home visit. Participants are instructed on how to take the supplement and given appropriate contact information to use for questions or to alert study personnel of any potential adverse events. Each participant receives a bottle labeled only with a group-level identification number unique to randomization group (for which the master study code is accessible only through a locked cabinet by a study coordinator), directions for use, primary research site name, batch number, and IRB protocol number. Participants are asked to record any changes in symptoms and medication or nutritional supplement use in a daily diary using a form reviewed at each study time point.

The intervention and placebo capsules are from the same lot. The placebo utilized in this clinical trial is an inactive dietary supplement composed of cellulose, microcrystalline cellulose, silicon dioxide, and vegetable stearate, and formulated by the manufacturer to be as similar as possible to the active intervention in appearance and other key characteristics. In an attempt to match scent, both active intervention and placebo will have their lid undersides swabbed with a cotton ball containing one drop of cinnamon essential oil (steam-distilled *Cinnamomum zeylanicum* leaf). Packaging for the control group is identical to packaging for the treatment group.

### Randomization and blinding

Treatment assignment is randomly assigned, in a 1:1 ratio, at each site using a block size of 6. The block randomization sequence schedule is password-controlled by an appointed research team member not involved in the screening or enrolling of participants. The treatment assignments are known to the study coordinator who maintains the allocation master list in a sealed, opaque envelope. Participants, treating clinicians, and outcome assessors are blinded to intervention group status.

### Sample size

The research team aims for a final sample of 40 participants with prediabetes in this clinical trial. Participants are recruited from within clinical practices, mobile health screenings conducted as part of a separate non-intervention study [106], support groups, and health fairs, with 20 participants allocated to the active group (GlucoSupreme™ Herbal) and 20 participants allocated to the control group (placebo). In order to account for a conservative and higher-than-expected dropout rate of 10%, 44 participants will be enrolled to achieve the target sample size. The sample size of 44 participants was estimated by assuming 80% statistical power, a significance level (alpha) of 0.05 using a two-sided, two-sample, equal variance *t* test, an anticipated difference in group means of 0.91 standard deviations for continuous variables, and 10% attrition. The sizes of these treatment effects are large (i.e., between-group differences will need to be considerable in order for the treatment to demonstrate statistically significant improvements relative to placebo).

This study will utilize an adaptive sample size re-estimation (SSR) approach [107] where participant enrollment may be extended beyond the originally planned sample size if interim effect size is smaller than anticipated, but still promising, thereby preserving study power. The adaptive SSR technique will include: (1) examination of available unblinded endpoint data by the study statistician after 30 subjects are enrolled, (2) calculation of conditional power (CP), and (3) sample size modification in order to maintain study power, if required, based on pre-defined limits. CP is defined as the power of rejecting the null hypothesis at the end of the trial conditional on the observed data accumulated up to the time of the planned interim analysis. CP estimates will be categorized into one of three “zones”— Favorable, Promising, or Unfavorable. The Favorable zone will be defined as CP ≥ 80%. CPs in the Favorable zone will require no sample size adjustment (*n* = 40). Similarly, CP estimates that fall into the Unfavorable zone (CP < 50%) will require no sample size modifications (*n* = 40). However, if CP falls into the Promising zone (CP between 50 and 79%), the sample size will be recalculated such that CP equals the originally planned statistical power (i.e., 80%), up to a maximum of 66 subjects. This methodology preserves the overall type I error rate, no adjustments to alpha levels or confidence intervals are necessary, and standard statistical tests can be used for data analysis. Should the trial need to utilize adaptive SSR and enroll to the maximum sample size of 66, a difference in group means of 0.74 standard deviations could be detected.

Participants will be withdrawn from the study if they wish to withdraw or experience serious adverse events (SAEs) for any reason. If SAEs are reported, the site physician and principal investigator of the study will be contacted by the study coordinator, the participant will be withdrawn from the study, and a treatment plan will be implemented. Any dropouts will be included in the statistical analysis as intention-to-treat (ITT) using the last value carried forward (LOCF) method. Subsequent analyses will also be conducted on the per-protocol population which fully completes the study in order to determine if there are any discernible differences in these populations for the purposes of more broadly extrapolating the generalizability of results.

### Follow-up

#### Data collection points

**Fig. 1:**
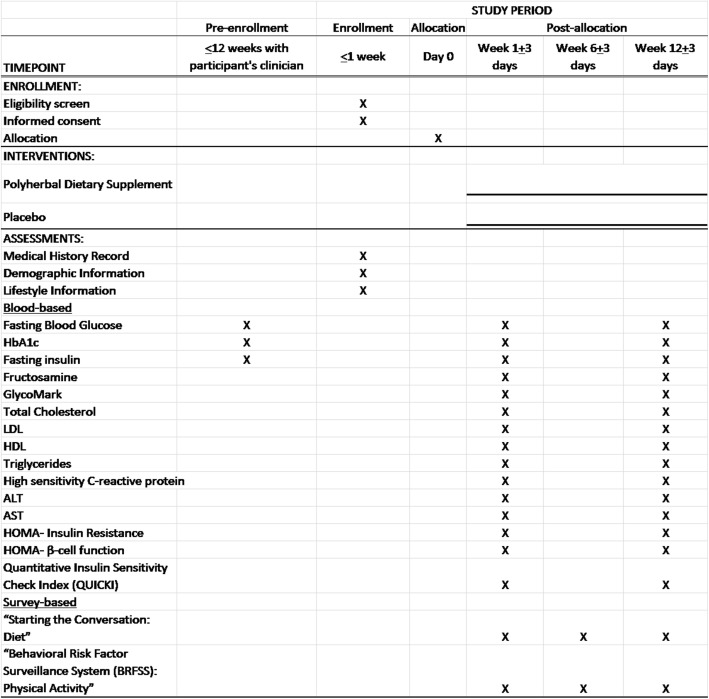
Data collection points for a polyherbal supplementation intervention for prediabetic adults

All data collection points are displayed in Fig. 1, based on the Standard Protocol Items: Recommendations for Interventional Trials (SPIRIT) guideline [108].

- Screening period: +/- 3 days before the intervention
- Intervention period: 12 weeks
- Follow-up period: none

### Outcome measures

Outcome measures include glycemic parameters, lipids, inflammation, and progression to T2D (Table 2). A number of glycemic parameters in addition to fasting blood glucose and HbA_1c_ are measured throughout this study. Because insulin resistance often sets the stage for progression to T2D by placing a high demand on insulin-producing (β) beta-cells during a prediabetic state, we are using the validated HOMA-IR to estimate insulin resistance (calculated as: (Fasting insulin [μU/L] × Fasting blood glucose [nmol/L])/22.5 [109]). Likewise, the validated Quantitative Insulin Sensitivity Check Index **(**QUICKI) measurement to assess insulin sensitivity has also been used extensively, including in herbal dietary supplement clinical trials, and is (calculated as: 1/Log-fasting blood glucose [mg/dL] + Log-fasting insulin [μU/mL]) [109]. Additionally, pancreatic β-cells produce insulin. Thus, participant HOMA-β is being estimated as well (calculated as: (20 × Fasting insulin [μU/L])/(Fasting blood glucose [mmol/L] – 3.5) [109]).

**Table 2:** Primary and secondary outcomes

Endpoint events	
Primary outcomes	
Glycemic parameters	Normal range reference
Fasting blood glucose	< 100.9 mg/dL
Glycosylated hemoglobin/HbA_1c_	< 5.7%
Fructosamine	200–285 μmol/L [63]
GlycoMark	~ 180 mg/dL
Fasting insulin	< 25 μU/L
Absence of high HOMA-Insulin Resistance	General US adult population: < 2.73 vs. ≥ 2.73 [63]
Quantitative Insulin Sensitivity Check Index	~ 100% [66]
Pancreatic β-cell function	~ 100% [66]
Secondary outcomes	
Lipid profile	
Total cholesterol	≤ 19 years: < 170 mg/dL; ≥ 20 years: 125–200 mg/dL
Low-density lipoprotein	< 100 mg/dL
High-density lipoprotein	≤ 19 years: > 45 mg/dL; ≥ 20 years men: ≥ 40 mg/dL; ≥ 20 years women: ≥ 50 mg/dL [129]
Triglycerides	< 150 mg/dL [130]
Inflammation	
High-sensitivity C-reactive protein (hs-CRP)	Varies^a^
Progression to type-2 diabetes (T2D) (according to 2016 ADA diagnostic criteria [39, 40])	

*ADA* American Diabetes Association*, HbA*_*1c*_ glycated hemoglobin/HbA1c, *HOMA-IR* homeostatic model of Insulin Resistance, *US* United States

^a^Elevated CRP levels indicate increased risk of T2DM [131] with normal CRP range 0.0–5.0 mg/L [132], while hs-CRP reference values applicable to cardiovascular risk assessment are < 2.0 mg/L [133]

Other relevant glycemic parameters required to gain a well-rounded understanding regarding potential impacts of the intervention include fructosamine and GlycoMark. Fructosamine is the concentration of plasma glucose over the lifetime of the protein, and represents the degree of glycation in many serum proteins involved in glucose synthesis. It is a test utilized in clinical practice and clinical studies of natural products [31, 110], is often viewed as an adjunct to HbA_1c_ (a measurement which is recommended every 3 months in clinical practice and reflects longer-term glycemic change over several months [34]) and fasting glucose measurements (short-term glycemic change), and reflects intermediate-term (previous 2–3 weeks) glycemic change. GlycoMark, a blood test used in clinical practice to determine peak hyperglycemia, provides accurate recognition of recent glycemic deterioration or improvement (within the previous 1–2 weeks) [111–113].

#### Safety indices

- Alanine aminotransferase (ALT) levels to assess hepatic function
- Aspartate aminotransferase (AST) levels to assess hepatic function

Both safety indices will be tested on visit 1 (baseline visit) and visit 2 (at 12 weeks).

#### Additional indicators

- Incidence of T2D, calculated using the following formula:
  $$ \mathrm{Incidence}\ \mathrm{of}\ \mathrm{diabetes}\ \mathrm{mellitus}=\mathrm{Cases}\ \mathrm{of}\ \mathrm{diabetes}\ \mathrm{mellitus}\ \mathrm{at}\ \mathrm{end}\ \mathrm{of}\ \mathrm{study}/\mathrm{Total}\ \mathrm{cases}\ \mathrm{of}\ \mathrm{each}\ \mathrm{group}\times 100\% $$
- Reversion rates of each continuous parameter after 12 weeks (including reversion from prediabetes to normal glucose tolerance), calculated using the following formula:
  $$ \mathrm{Reversion}\ \mathrm{rate}=\mathrm{Cases}\ \mathrm{of}\ \mathrm{parameter}\ \mathrm{that}\ \mathrm{have}\ \mathrm{returned}\ \mathrm{to}\ \mathrm{normal}\ \mathrm{range}/\mathrm{Cases}\ \mathrm{of}\ \mathrm{each}\ \mathrm{group}\times 100\% $$

### Adverse event monitoring

#### Reporting of adverse events

At enrollment, participants are given contact information that they may use anytime (24 h a day/7 days per week) to report potential adverse events (AEs), which may include a physical, psychological, or laboratory event, or an increase in the severity or frequency of a pre-existing symptom or condition. Serious adverse events (SAEs) are defined by the International Conference on Harmonization of Technical Requirements for Registration of Pharmaceuticals for Human Use (ICH) as life-threatening, death, inpatient hospitalization/prolongation of existing hospitalization, congenital anomaly, persistent or significant disability/incapacity, and requirement of an intervention to prevent permanent impairment/damage [114, 115].

#### Documentation of adverse events

Participants are also informed at enrollment of their requirement to immediately report AEs/SAEs to the research team if and when they occur, and the research team will inquire about AEs/SAEs during telephone calls or home visits at weeks 6 and 12. Following AE/SAE reports, an AE form will be completed and reported to the clinic site physician and principal investigator. The contents of each form are reviewed for clarity by the principal investigator. Information regarding the time of event, severity, duration, and remedial actions (i.e., discontinuation of study supplement, physician follow-up, and treatment administered, if necessary) are documented on each AE form.

Participants who experience SAEs will be immediately withdrawn from the trial and these events will be reported to the principal investigator and clinic site physician within 24 h. The SAE will subsequently be reported to the U.S. Food and Drug Administration (FDA) Adverse Event Reporting System (AERS) and Natural MedWATCH, a tool developed and maintained by the Therapeutic Research Center’s Natural Medicines Comprehensive Database (https://naturaldatabase.therapeuticresearch.com/nd/adverseevent.aspx?s=ND&cs=naturalstandard) regardless of perceived causality to study intervention, followed by a report to the FDA including AEs not initially deemed reportable if the follow-up information causes a change in assessment. In accordance with Good Clinical Practice (GCP) guidelines [116], the informed consent form (ICF) will also be revised when new information becomes available that may be relevant to the participant’s consent, including the addition of new AEs related to the study treatment.

#### Relationship between adverse events and study treatments

Researchers will determine the potential causality between the study supplement and reported AEs/SAEs using the following criteria to determine severity and relatedness:

- Whether the suspected AE/SAE appears after treatment administration, in addition to history of symptom(s) associated with the suspected AE/SAE
- Whether the suspected AE/SAE belongs to known or suspected potential adverse reactions of the study supplement; these include: dizziness, skin rash, headache, tinnitus, hyper/hypotension, and gastrointestinal events
- Whether the AE/SAE dissipated or disappeared after discontinuation of the study supplement
- Whether a participant’s health history or co-occurring medicine or supplement use may be responsible for the AE/SAE (to be determined by the principal investigator and/or study physicians)

#### Criteria for the evaluation of safety

The safety of the study supplement will be assessed as relatively safe without any, or minimal, changes in hepatic function (ALT, AST).

In addition, nausea, vomiting, loss of appetite, fatigue and weakness, sleep problems, changes in urination, decreased mental sharpness, muscle twitches and cramps, swelling of feet and ankles, persistent itching, chest pain, shortness of breath, and uncontrollable high blood pressure [117–120] will be assessed via the reporting of all AEs/SAEs. The study physicians will determine potential suspension of study supplement, if necessary, and will follow-up with all participants reporting SAEs and attempt to perform medical evaluation to determine the cause of the SAE and its possible relationship to the study intervention.

### Statistical analysis plan

#### Analysis software

All parameters will be analyzed using SAS v9.4 (Cary, NC, USA).

#### Analysis of datasets

- Full analysis of ITT: the LOCF method will be used for missing data points
- Per-protocol set (PPS): PPS analyses will include participants who meet the following characteristics: meet inclusion criteria, are between 75 and 107% compliant with dosing per self report [121], have complete laboratory assessments, have primary outcome measurements within the study timeline
- Safety set (SS): the incidence of reported AEs/SAEs will be identified for all documented safety outcomes; biomarkers will be assessed according to laboratory test results

### Statistical analysis methods

The number of participants screened, randomized, withdrawn early, and completing the study will be tabulated by treatment group and presented in a Consolidated Standards of Reporting Trials (CONSORT)-herbal diagram [122]. Reasons for early withdrawal, if any should occur, will be presented.

Descriptive statistics will be computed for baseline and demographic characteristics and tabulated by treatment group, and may include means, medians, standard deviations, inter-quartile ranges, and percentages, as dictated by the form of each variable. Independent *t* tests will be used for group comparisons (polyherbal dietary supplement group versus control group) on primary and secondary outcomes for each time point. Data transformations may be performed in the event of non-normally distributed data, or the Wilcoxon-Mann-Whitney non-parametric test will be used to assess differences between the means of outcomes between both groups. Fisher’s exact chi-square will be used to assess differences in categorical variables by group, in order to establish whether additional possible influences of dietary intake, broad differences in physical activity, or other demographic differences between study groups may accompany changes in any of the outcomes of interest. The chi-square test will be used for comparing additional endpoint events (e.g., incidence of T2D).

Study outcomes over the 12-week supplementation period will be analyzed using a mixed-model analysis of variance for continuous variables, which controls for within-subject and between-subject correlation. Repeated measures analysis of variance and logistic regression will be used to evaluate potential changes in physical activity and dietary intake, in order to determine whether these differ by treatment group to potentially influence observed effects of the intervention. If applicable, the proportion of subjects reporting at least one AE will be tabulated in each treatment group and compared using Fisher’s exact test. Statistical significance will be set at *p* < 0.05. No adjustments for multiplicity will be performed. The primary analysis will be conducted on the ITT population. Per-protocol and sensitivity analysis may be conducted as appropriate.

### Document conservation and summary

Data to be included in this study includes, but is not limited to, demographic information, medical history, current medication(s), study compliance, study outcomes listed previously, AEs, participant withdrawals, and a screener to detect overall changes in dietary behavior and physical activity. After electronic data entry, all data is stored on secure University of Maryland School of Medicine (UMSOM) servers. All study and source documents, including clinic agreements, consent forms, and signatures of study participants, will be retained by researchers for a period of at least 6 years post study completion in a secure place [116]. Only approved study staff will have access to the data.

### Trial management

#### Management of protocols

All study personnel are trained to provide consistent dissemination of study materials and information to participants, and have undergone recent CITI training for Clinical Research (https://about.citiprogram.org/en/homepage/). All researchers, physicians, and related personnel are required to understand and adhere to protocol details and each must have the capability to explain the study process to anyone, including participants. Any changes in protocol will be subject to additional IRB amendment approval.

#### Measures for compliance of participants

Participant compliance is tracked and examined by researchers. The research team verifies that participants have received the study supplement within the predetermined window of time, explains the proper dosage at baseline, and ensures that participants are consuming the expected number of capsules at their 6-week telephone call or visit. The daily diary is checked by researchers at the end of a participant’s duration in the study in addition to pill counts to determine adherence to the study supplement.

#### Monitoring and inspection

Participant data is entered into an Excel spreadsheet within one week of being collected, and is assessed for integrity and consistency by members of the research team in the following order: the study coordinator, the principal investigator of the study, and statistical sub-investigators for the study. The Standard Protocol Items: Recommendations for Interventional Trials (SPIRIT) Checklist is available in Additional file 1.

#### Bias control

All dietary supplements and medications taken by participants during the study period are recorded and analyzed at the conclusion of the study. The daily use of oral hypoglycemic medications or insulin injections during the study period is considered an exclusion criterion during screening and prohibited during the study, resulting in exclusion from the per-protocol analysis, should it occur. Broad changes in dietary intake and physical activity are recorded at each time point in order to account for potential bias.

## Discussion

Over 50% of American adults take a dietary supplement for a variety of health-related goals [123]. Glycemic control is a common goal of dietary supplementation, since prediabetes is an increasingly common risk factor for T2D and pharmacological agents are currently limited in the treatment of prediabetes. There is a pressing need to provide evidence-based options to improve glycemic control so as to prevent the burden of T2D, for which costs have increased 41% between 2007 and 2012 in the United States [124]. The Diabetes Prevention Lifestyle Change Program (DPP) delayed T2D incidence in a multicenter randomized controlled trial in the United States. by 58% compared to metformin (31%) over a 4-year period [125]. Further, 15-year follow-up demonstrated DPP participants continued to delay the T2D development by 27% compared to placebo, superior to metformin (18% delay) [126]. Additional beneficial approaches to delay or prevent T2D are needed for people unable to implement broad lifestyle interventions, such as the DPP, or to perhaps supplement these lifestyle changes. Rigorous evaluation of the safety and efficacy of commercially available, polyherbal dietary supplements appears warranted, as these supplements are popular and may be more representative of traditional and modern herbal medicine practice than single herbs in isolation [82, 127]. In light of the evidence supporting the use of some herbal dietary supplement formulations to improve glycemic parameters, the study investigators hypothesize that the polyherbal dietary supplement under study may restore glycemic parameters to normal ranges, irrespective of broad changes in diet or physical activity. Thus, this polyherbal formulation may ultimately be helpful in deterring the incidence of T2D by preventing glycemic parameters from reaching ranges associated with this costly disease. The randomized, double-blinded design is utilized in this study to gain an understanding of the safety and efficacy of this polyherbal dietary supplement on 12-week changes in short-term, medium-term, and comparatively longer-term markers of glycemic function, as measured by validated laboratory assessment of fasting blood glucose and fasting insulin (short-term), fructosamine (medium-term), and HbA_1c_ (longer-term). In addition, we measure a number of clinically relevant outcomes that capture peak glycemic dysfunction (GlycoMark), insulin resistance, β-cell function, insulin sensitivity (QUICKI), lipid profile (total cholesterol, low-density lipoprotein (LDL), HDL, and triglycerides), inflammation (high-sensitivity C-reactive protein (hs-CRP)), and progression to T2D.

Although the study design is optimized by the selection of a study treatment containing only plant ingredients with demonstrated mechanisms of action (i.e., enzymatic, signal enhancement, etc.) [40, 43, 44, 62–92, 128], the generalizability of this study cannot be extended to smokers since they were excluded for potential safety concerns. In addition, the small sample size of this proof-of-concept and exploratory study confers a limited scope to examine potential adverse effects, and the possibility remains that investigators may find smaller than anticipated effects regarding outcomes. This may pose a potential limitation in drawing conclusions regarding outcomes. However, the study is designed to detect considerable between-group differences, determined a priori through power analyses and the accommodation of an optional use of adaptive sample size re-estimation. If the planned trial shows beneficial effects on the outcomes, subsequent trials should ideally utilize a larger sample and examine potential longer-term (> 12-week) effects of the study treatment on glycemic control.

### Trial status

The trial has been approved by the University of Maryland School of Medicine IRB (#HP-00075768) and Western IRB (#20171220) and retroactively registered at ClinicalTrials.gov (NCT03388762, https://clinicaltrials.gov/ct2/show/NCT03388762) on 4 January 2018. At the time of submission, this trial was actively recruiting participants.

## Additional file

Additional file 1: Data collection points for a polyherbal supplementation intervention for prediabetic adults. (PNG 67.6 kb)

## Abbreviations

AEs: Adverse events
CP: Conditional power
FBG: Fasting blood glucose
FDA AERS: U.S. Food and Drug Administration Adverse Event Reporting System
GCP: Good Clinical Practice
HbA_1c_: Glycated hemoglobin/A1c
HDL: High-density lipoprotein
HOMA-IR: HOMA – Homeostatic model of Insulin Resistance
HOMA-β: HOMA – Homeostatic model of β-cell function
hs-CRP: High-sensitivity C-reactive protein
ICF: Informed Consent Form
IRB: Institutional Review Board
ITT: Intention-to-treat
LDL: Low-density lipoprotein
LOCF: Last observation carried forward
PPS: Per-protocol set
QUICKI: Quantitative Insulin Sensitivity Check Index
SAEs: Serious adverse events
SPIRIT: Standard Protocol Items: Recommendations for Interventional Trials
SS: Safety set
SSR: Sample size re-estimation
T2D: Type 2 diabetes
UMSOM: University of Maryland School of Medicine
