# Supplementary figures and images for: Polyherbal dietary supplementation for prediabetic adults: study protocol for a randomized controlled trial
Source: Trials. 2019 Jan 7;20:24. doi: 10.1186/s13063-018-3032-6 (PMC6323847; doi:10.1186/s13063-018-3032-6)

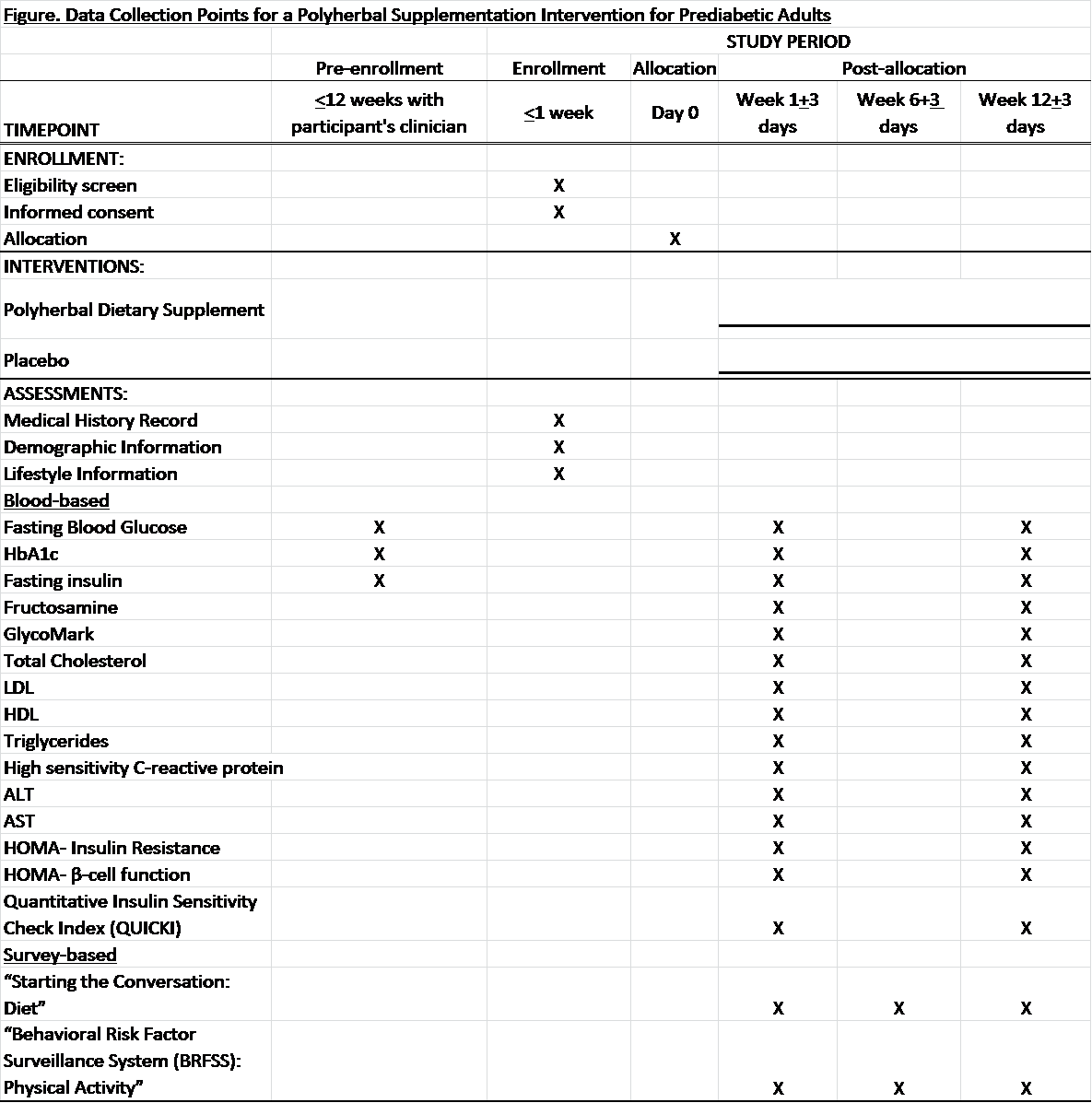

Supplement: Supplementary file 1 — Data collection points for a polyherbal supplementation intervention for prediabetic adults. (PNG 67.6 kb) [file 13063_2018_3032_MOESM1_ESM.png]
